# 31.4 GENETIC, IMMUNOLOGICAL AND BIOCHEMICAL MARKERS OF TREATMENT RESPONSE IN SCHIZOPHRENIA

**DOI:** 10.1093/schbul/sby014.130

**Published:** 2018-04-01

**Authors:** Marion Leboyer, Stephane Jamain

**Affiliations:** 1 AP-HP, Université Paris-est-Créteil; 2 Inserm, Université Paris-Est-Créteil

## Abstract

**Background:**

One of the major shortcomings in the current treatment of schizophrenia is that we have no valid criteria in clinical practice to decide which antipsychotic treatment should be chosen first. This is why we need to define a blood-based biological signature of treatment response that can be easily tested at patient bedside and would also help to identify molecular mechanisms of treatment response by determining biological changes associated with symptom improvements.

**Methods:**

Through a European consortium on Optimization of Treatment and Management of Schizophrenia in Europe (FP7, OPTiMiSE), we conducted a clinical trial on treatment response with Amisulpride in 500 subjects with first episode psychosis. For each patient, biological samples (DNA, RNA, plasma and serum) have been collected before treatment and during follow-up visits at weeks 4, 10 and 22, to measure biological changes associated with treatment initiation and with symptoms improvements. We combined multiple high-throughput technologies for transcriptome, genome, metabolome, proteome analyses before and after treatment.

**Results:**

The transcriptome analysis conducted on 10,683 genes expressed in peripheral mononuclear cells identified significantly more genes differentially expressed after treatment in 112 patients who will be in remission after 4-weeks treatment than in 51 non-remitters. Using interaction network analysis, we identified biological pathways affected by Amisulpride. For some genes, the expression level was significantly correlated with symptom improvement. Moreover, some genes were already differentially expressed before treatment between remitters and non-remitters, suggesting they might be used to predict treatment outcome. In addition, we identified genetic variations associated with gene expression level and thus may explain individual difference in treatment response.

In parallel, as recent biological data have suggested a preponderant role of innate and adaptive immune system in the vulnerability to schizophrenia or in antipsychotic treatment response (Fond et al, 2015), we paid a particular attention to the analysis of inflammatory markers and the presence of auto-antibodies in patients’ sera. Circulating autoantibodies against glutamatergic N-methyl-D-aspartate receptor (NMDAR-Ab) have been reported in up to 10% of patients with psychotic disorders. In our study, we demonstrated the advantage of using cutting-edge methods to ascertain the presence of NMDAR-Ab in seropositive patients that cannot be clinically identified ((Jezequel et al, 2017). Indeed, the only clinical characteristics found in NMDAR-Ab seropositive patients, was the high frequency of female patients, the presence of mild neurologic symptoms and signs of antipsychotic intolerance. In addition, using an advanced statistical classification algorithm, we defined clinically-based subgroups of patients who had specific cytokine signature associated to remission after 4-weeks of treatment, suggesting that these markers may be used to predict treatment response.

**Discussion:**

Altogether, our multilevel biological approach resulted in the identification of promising biomarkers, which may be used both to predict drug response and remission in first psychosis episode.

